# Advancing Public Health Entrepreneurship to Foster Innovation and Impact

**DOI:** 10.3389/fpubh.2022.923764

**Published:** 2022-05-27

**Authors:** Terry T. K. Huang, Alessandro Ciari, Sergio A. Costa, Teresa Chahine

**Affiliations:** ^1^Center for Systems and Community Design, NYU-CUNY Prevention Research Center and Firefly Innovations, Graduate School of Public Health and Health Policy, City University of New York, New York, NY, United States; ^2^School of Management, Yale University, New Haven, CT, United States

**Keywords:** public health entrepreneurship, public health innovation, impact investment, sustainability, ESG

## Introduction

The magnitude of challenges facing public health today is daunting, as illustrated by the ambitious United Nations Sustainable Development Goals (UN SDGs). For example, despite billions of dollars of investment, obesity, as a major contributor to leading non-communicable diseases (NCDs) such as cardiovascular disease, diabetes, respiratory disease and certain cancers, continues to increase worldwide. In the U.S. alone, the prevalence of adult obesity grew from 30.5% in 1999–2000 to 42.4% in 2019–2020 (1). In addition, obesity now affects over 20% of U.S. children, a significant increase from prior decades despite expert beliefs in recent years that childhood obesity had perhaps reached a plateau (2). Worst yet, even in places where childhood obesity has shown a significant decline as a result of aggressive multi-pronged policies and interventions, obesity disparities among minority and disadvantaged populations have actually widened (3). At the same time, it is also the case that the vast majority of public health research and development is not implemented and scaled to meet the urgency of the challenge. It is estimated that it takes 17 years to scale up 14% of public health innovations, due to a great extent to the limitations presented by the traditional grant-based system for public health research and development (4). All of this suggests that we need a more agile, dynamic system to foster and scale innovation in public health solutions. In recent years, there have been emerging calls for attention to the incorporation of entrepreneurship methods in public health education, research and practice (5). Public health entrepreneurship can be defined as a continuous mission- and innovation-driven process to create new ways of tackling public health challenges and to produce lasting social or systems change (5). In this opinion piece, we propose additional arguments for why the time is now for investment in public health entrepreneurship on part of public, private and academic sectors.

That public health disparities, including those related to obesity and diet-related diseases, persist as a policy-resistant problem is, in part, a function of the fact that public health research and practice to date remain top-down. Rarely do we innovate from the perspective of true community values and needs (6). Yet, lessons from innovations based on human- and community-centered design suggest that there is an opportunity to leverage bottom-up and asset-based approaches to public health challenges (7). One strategy aligned with this thinking is to harness and cultivate untapped innovations in communities. Such innovations are most often generated by individuals with lived experience of the problems they seek to solve – a key missing ingredient in the startup ecosystem today. For example, six national surveys (U.S., Canada, U.K., Japan, Finland, and South Korea) of household end-users found a prevalence of user innovators between 1.5 and 6.1% across nations (8–13). Within this broad group, 2 to 8% of user innovators (~1 million individuals) focused on medical or health-related product innovations. These numbers do not include service or behavioral interventions, suggesting even more untapped innovations. In another study among patients with a broad range of health challenges, 53% of patients came up with new solutions to cope with their conditions; 15% of these innovations were completely novel as evaluated by medical experts while the remainder represented re-invention of existing solutions (14). In this study, both patients and caregivers reported that their quality of life improved by ~1.4–2.0 points on a 7-point scale, pointing to the potentially significant public health impact these user-generated innovations hold if scaled up. We argue that crowdsourcing and supporting these community-driven innovations present a unique opportunity to address health disparities.

Public health entrepreneurship is urgent and timely. According to MSCI ESG Research (ESG refers to environmental, social and governance), over $30 billion of wealth is expected to pass hands from baby-boomers to millennials in the coming decades (15). Among millennial wealth owners, 95% are interested in impact investing and 57% have intentionally stopped investing because of a company's negative impact on people's health and wellbeing (15). Impact investment is defined by the intentionality, measurability and accountability of investing – areas that few can do better than those in public health. Beyond impact investing, there is a dire need for public health expertise in the private sector, including in the venture world, in light of growing demand for meaningful ESG management, as public health experts are exceptionally trained in social and environmental determinants of health, health equity, and impact monitoring and evaluation. Additionally, with regards to the daunting cross-sectoral challenges facing public health today, including NCDs, emerging pandemics, and climate change, the private sector offers unique expertise to help solve public health problems. The COVID-19 pandemic serves as a good example: without the agility of public-private partnerships, it would have been impossible to deliver an effective vaccine in 9 months based on new technology (16). The key now is to build on this recent experience and extend this innovation approach to many more areas of public health. By bringing together the public, and private sectors, public health entrepreneurship mobilizes new resources including talent and capital.

There are best practices for engaging in and teaching public health entrepreneurship (17). Rooted in human- and community-centered design, public entrepreneurship entails developing a deep understanding of the true needs or pain points of a community and undertaking an iterative cycle of ideating, prototyping and testing potential solutions. As part of this process, rapid and frequent failures are paramount to arriving at true innovation in the end, contrary to the conventional approach to public health research that emphasizes the likelihood of success (in order to obtain funding, for example). In addition, business modeling is integral to this process, as user/community fit must be accompanied by a viable and scalable model of revenue generation and cost-effective operation, including clarity about supply chain logistics and other stakeholder actions. Revenue generation is key, for even not-for-profit organizations must generate resources to achieve sustainability. To incorporate entrepreneurship into public health education, public health schools should consider expanding course offerings to encompass design, marketing, business development and operations, finance, and software development and other technologies (18).

At the City University of New York Graduate School of Public Health and Health Policy, a unique public health entrepreneurship platform called Firefly Innovations (https://firefly-innovations.com) was launched in early 2020. The goal of Firefly Innovations is to identify, cultivate and accelerate impact-driven public health ventures, particularly those that are led by founders with lived experience of the problems they aim to solve. To date, Firefly Innovations has hosted two “designathons” (akin to hackathons without necessarily computer coding) and two 10-week summer accelerators focused on health equity. The first accelerator in Summer 2020 was focused on the COVID-19 pandemic. Because of the emphasis on health equity, Firefly Innovations organically draws in ventures that are aligned with its mission. Thus far, Firefly Innovations has received more than 150 solutions from entrepreneurs based in 16 different countries. Of the more than 30 ventures that have been actively supported or accelerated, 100% of them have at least one minority founder and 60% are women-led. Throughout this effort, Firefly Innovations also grew a network of more than 200 collaborators, including speakers, mentors, judges and expert advisors. It has also successfully introduced educational curricula including design thinking in public health and hands-on public health entrepreneurship/venturing within public health graduate degree programs. In addition to the accelerator and university curricula, lectures and workshops are organized regularly to converge like-minded thinkers and actors from diverse public and private sectors with public health researchers and students.

The field of public health is at a momentous juncture with the opportunity to go beyond traditional public health academia and act more strategically across sectors to foster and scale public health innovations. We must do so if we are to meet the challenges presented by the UN SDGs. Increasingly, public health students are demanding action-oriented training, research and practice (19). As public health educators, we also have a duty to ensure that our graduates are ready for the rapidly changing landscape of technology and are equipped to assume leadership in devising solutions for the world's complex problems of today and tomorrow. The integration of public health and the entrepreneurial sector is critical to building a healthy and sustainable world. The time to invest in public health entrepreneurship is now.

## Author Contributions

TH produced the structure of the opinion and wrote the first draft of the manuscript. AC wrote parts of the manuscript. TC and SC edited the manuscript. All authors contributed to the article and approved the submitted version.

## Funding

TH and AC were partially supported by a grant (U48DP006396) from the Centers for Disease Control and Prevention.

## Conflict of Interest

The authors declare that the research was conducted in the absence of any commercial or financial relationships that could be construed as a potential conflict of interest.

## Publisher's Note

All claims expressed in this article are solely those of the authors and do not necessarily represent those of their affiliated organizations, or those of the publisher, the editors and the reviewers. Any product that may be evaluated in this article, or claim that may be made by its manufacturer, is not guaranteed or endorsed by the publisher.
